# Supplementary material for: Overexpression of fucosyltransferase 8 reverses the inhibitory effect of high-dose dexamethasone on osteogenic response of MC3T3-E1 preosteoblasts
Source: PeerJ. 2021 Dec 9;9:e12380. doi: 10.7717/peerj.12380 (PMC8667747; doi:10.7717/peerj.12380)
Supplement: Supplemental Information 1 [file peerj-09-12380-s001.zip › Supplementary Materials 20210719/Statistical Data.docx]

**Figure 1A:**

|  | F | P value |
| --- | --- | --- |
| Time of interventoin  x Dex concentration | 35.56 | <0.0001 |
| Time of interventoin | 149.8 | <0.0001 |
| Dex concentration | 500.1 | <0.0001 |
| Subject | 0.5384 | 0.8523 |
| Residual |  |  |

| Dex concentration  (Compare that to 0 M) | absorbance value OD | repeated measure | P Value |
| --- | --- | --- | --- |
| 1d | | | |
| 10^-5^ M | 0.5100±0.0082 | 3 | 0.0139 |
| 10^-6^ M | 0.5380±0.0070 | 3 | 0.0673 |
| 10^-7^ M | 0.6083±0.0032 | 3 | 0.1370 |
| 10^-8^ M | 0.6147±0.0074 | 3 | 0.0751 |
| 0 M | 0.5100±0.0147 | 3 |  |
| 2d | | | |
| 10^-5^ M | 0.5273±0.01267 | 3 | 0.0043 |
| 10^-6^ M | 0.5730±0.0056 | 3 | 0.0361 |
| 10^-7^ M | 0.6240±0.0056 | 3 | 0.1093 |
| 10^-8^ M | 0.6430±0.0056 | 3 | 0.0174 |
| 0 M | 0.6037±0.0095 | 3 |  |
| 3d | | | |
| 10^-5^ M | 0.5587±0.0045 | 3 | 0.0181 |
| 10^-6^ M | 0.5827±0.0047 | 3 | 0.0635 |
| 10^-7^ M | 0.6483±0.0068 | 3 | 0.0651 |
| 10^-8^ M | 0.6737±0.0087 | 3 | 0.0099 |
| 0 M | 0.6167±0.0120 |  |  |
| 7d | | | |
| 10^-5^ M | 0.3567±0.0250 | 3 | 0.0044 |
| 10^-6^ M | 0.3417±0.0388 | 3 | 0.0054 |
| 10^-7^ M | 0.7380±0.0452 | 3 | 0.0187 |
| 10^-8^ M | 0.7450±0.0272 | 3 | 0.0061 |
| 0 M | 0.5610±0.0340 |  |  |
| 14d | | | |
| 10^-5^ M | 0.5623±0.0391 | 3 | 0.0197 |
| 10^-6^ M | 0.5437±0.0424 | 3 | 0.0155 |
| 10^-7^ M | 0.9333±0.0246 | 3 | 0.0108 |
| 10^-8^ M | 0.9780±0.0373 | 3 | 0.0045 |
| 0 M | 0.7300±0.0417 |  |  |

**Figure 1B:**

**FUT8**

| ANOVA summary | |
| --- | --- |
| F | 534.9 |
| P value | <0.0001 |
| R square | 0.9953 |

| Dex concentration (M) (Compare that to 0 M) | mRNA relative expression | P Value |
| --- | --- | --- |
| 10^-5^ | 0.1233±0.005774 | <0.0001 |
| 10^-6^ | 0.2800±0.02000 | <0.0001 |
| 10^-7^ | 1.347±0.05508 | <0.0001 |
| 10^-8^ | 1.187±0.005774 | 0.0003 |
| 0 | 1.003±0.07095 |  |

**ALP**

| ANOVA summary |  |
| --- | --- |
| F | 40.19 |
| P value | <0.0001 |
| R square | 0.9414 |

| Dex concentration (M) (Compare that to 0 M) | mRNA relative expression | P Value |
| --- | --- | --- |
| 10^-5^ | 0.8867±0.005774 | 0.7209 |
| 10^-6^ | 0.7433±0.1102 | 0.1545 |
| 10^-7^ | 1.610±0.2750 | 0.0018 |
| 10^-8^ | 2.017±0.05774 | <0.0001 |
| 0 | 1.007±0.1328 |  |

**RUNX2**

| ANOVA summary | |
| --- | --- |
| F | 263.4 |
| P value | <0.0001 |
| R square | 0.9906 |

| Dex concentration (M) (Compare that to 0 M) | mRNA relative expression | P Value |
| --- | --- | --- |
| 10^-5^ | 1.217±0.1595 | 0.4476 |
| 10^-6^ | 0.8400±0.1473 | 0.6786 |
| 10^-7^ | 4.993±0.3365 | <0.0001 |
| 10^-8^ | 2.143±0.09074 | <0.0001 |
| 0 | 1.000±0.03606 |  |

**BMP2**

| ANOVA summary | |
| --- | --- |
| F | 42.62 |
| P value | <0.0001 |
| R square | 0.9446 |

| Dex concentration (M) (Compare that to 0 M) | mRNA relative expression | P Value |
| --- | --- | --- |
| 10^-5^ | 0.9067±0.1415 | 0.6278 |
| 10^-6^ | 0.6900±0.07550 | 0.0133 |
| 10^-7^ | 1.373±0.09292 | 0.0047 |
| 10^-8^ | 1.670±0.1127 | <0.0001 |
| 0 | 1.003±0.08145 |  |

**Osx**

| ANOVA summary | |
| --- | --- |
| F | 26.02 |
| P value | <0.0001 |
| R square | 0.9123 |

| Dex concentration(M) (Compare that to 0 M) | mRNA relative expression | P Value |
| --- | --- | --- |
| 10^-5^ | 0.9333±0.1060 | 0.9621 |
| 10^-6^ | 0.8833±0.06110 | 0.8462 |
| 10^-7^ | 2.297±0.3602 | 0.0001 |
| 10^-8^ | 1.783±0.1589 | 0.0046 |
| 0 | 1.020±0.2400 |  |

**Ocn**

| ANOVA summary | |
| --- | --- |
| F | 46.57 |
| P value | <0.0001 |
| R square | 0.9490 |

| Dex concentration(M) (Compare that to 0 M) | mRNA relative expression | P Value |
| --- | --- | --- |
| 10^-5^ | 0.8533±0.1570 | 0.7900 |
| 10^-6^ | 0.7667±0.1350 | 0.4663 |
| 10^-7^ | 2.343±0.3412 | <0.0001 |
| 10^-8^ | 2.310±0.2163 | <0.0001 |
| 0 | 1.000±0.01732 |  |

**PPARγ**

| ANOVA summary | |
| --- | --- |
| F | 29.64 |
| P value | <0.0001 |
| R square | 0.9222 |

| Dex concentration(M) (Compare that to 0 M) | mRNA relative expression | P Value |
| --- | --- | --- |
| 10^-5^ | 1.643±0.1686 | 0.0006 |
| 10^-6^ | 1.500±0.1646 | 0.0037 |
| 10^-7^ | 0.8467±0.1328 | 0.4777 |
| 10^-8^ | 0.6533±0.1106 | 0.0335 |
| 0 | 1.000±0.07937 |  |

**CEBPα**

| ANOVA summary | |
| --- | --- |
| F | 33.54 |
| P value | <0.0001 |
| R square | 0.9306 |

| Dex concentration(M) (Compare that to 0 M) | mRNA relative expression | P Value |
| --- | --- | --- |
| c | 1.657±0.1677 | 0.0001 |
| 10^-6^ | 1.617±0.1102 | 0.0002 |
| 10^-7^ | 0.8167±0.05508 | 0.1858 |
| 10^-8^ | 1.307±0.1301 | 0.0233 |
| 0 | 1.003±0.03055 |  |

**HDAC5**

| ANOVA summary | |
| --- | --- |
| F | 57.87 |
| P value | <0.0001 |
| R square | 0.9586 |

| Dex concentration(M) (Compare that to 0 M) | mRNA relative expression | P Value |
| --- | --- | --- |
| 10^-5^ | 2.153±0.1704 | <0.0001 |
| 10^-6^ | 1.330±0.1744 | 0.0462 |
| 10^-7^ | 0.8433±0.08737 | 0.4227 |
| 10^-8^ | 0.6433±0.1079 | 0.0255 |
| 0 | 1.007±0.1097 |  |

**Figure 1B Summary**

| Genes | Dex concentration(M) | | mRNA relative expression | P Value |
| --- | --- | --- | --- | --- |
| FUT8 | | 10^-5^ | 0.1233±0.005774 | <0.0001 |
|  | | 10^-6^ | 0.2800±0.02000 | <0.0001 |
|  | | 10^-7^ | 1.347±0.05508 | <0.0001 |
|  | | 10^-8^ | 1.187±0.005774 | 0.0003 |
|  | | 0 | 1.003±0.07095 |  |
| ALP | | 10^-5^ | 0.8867±0.005774 | 0.7209 |
|  | | 10^-6^ | 0.7433±0.1102 | 0.1545 |
|  | | 10^-7^ | 1.610±0.2750 | 0.0018 |
|  | | 10^-8^ | 2.017±0.05774 | <0.0001 |
|  | | 0 | 1.007±0.1328 |  |
| RUNX2 | | 10^-5^ | 1.217±0.1595 | 0.4476 |
|  | | 10^-6^ | 0.8400±0.1473 | 0.6786 |
|  | | 10^-7^ | 4.993±0.3365 | <0.0001 |
|  | | 10^-8^ | 2.143±0.09074 | <0.0001 |
|  | | 0 | 1.000±0.03606 |  |
| BMP2 | | 10^-5^ | 0.9067±0.1415 | 0.6278 |
|  | | 10^-6^ | 0.6900±0.07550 | 0.0133 |
|  | | 10^-7^ | 1.373±0.09292 | 0.0047 |
|  | | 10^-8^ | 1.670±0.1127 | <0.0001 |
|  | | 0 | 1.003±0.08145 |  |
| Osx | | 10^-5^ | 0.9333±0.1060 | 0.9621 |
|  | | 10^-6^ | 0.8833±0.06110 | 0.8462 |
|  | | 10^-7^ | 2.297±0.3602 | 0.0001 |
|  | | 10^-8^ | 1.783±0.1589 | 0.0046 |
|  | | 0 | 1.020±0.2400 |  |
| Ocn | | 10^-5^ | 0.8533±0.1570 | 0.7900 |
|  | | 10^-6^ | 0.7667±0.1350 | 0.4663 |
|  | | 10^-7^ | 2.343±0.3412 | <0.0001 |
|  | | 10^-8^ | 2.310±0.2163 | <0.0001 |
|  | | 10^-5^ | 0.8533±0.1570 | 0.7900 |
|  | | 0 | 1.000±0.01732 |  |
| PPARγ | | 10^-5^ | 1.643±0.1686 | 0.0006 |
|  | | 10^-6^ | 1.500±0.1646 | 0.0037 |
|  | | 10^-7^ | 0.8467±0.1328 | 0.4777 |
|  | | 10^-8^ | 0.6533±0.1106 | 0.0335 |
|  | | 10^-5^ | 1.643±0.1686 | 0.0006 |
|  | | 0 | 1.000±0.07937 |  |
| CEBPα | | 10^-5^ | 1.657±0.1677 | 0.0001 |
|  | | 10^-6^ | 1.617±0.1102 | 0.0002 |
|  | | 10^-7^ | 0.8167±0.05508 | 0.1858 |
|  | | 10^-8^ | 1.307±0.1301 | 0.0233 |
|  | | 10^-5^ | 1.657±0.1677 | 0.0001 |
|  | | 0 | 1.003±0.03055 |  |
| HDAC5 | | 10^-5^ | 2.153±0.1704 | <0.0001 |
|  | | 10^-6^ | 1.330±0.1744 | 0.0462 |
|  | | 10^-7^ | 0.8433±0.08737 | 0.4227 |
|  | | 10^-8^ | 0.6433±0.1079 | 0.0255 |
|  | | 10^-5^ | 2.153±0.1704 | <0.0001 |
|  | | 0 | 1.007±0.1097 |  |

**Figure 2B:**

**ALP**

| F test to compare variances | | |
| --- | --- | --- |
| sh vs.NC | F | 10.59 |
|  | P value | 0.1726 |
| sh+10^-6^ vs.10^-6^ | F | 2.039 |
|  | P value | 0.6581 |
| sh+10^-5^ vs.10^-5^ | F | 29.18 |
|  | P value | 0.0663 |

| Dex concentration (M) | plasmid | mRNA relative expression | P Value |
| --- | --- | --- | --- |
| 0 | NC | 1.003±0.07572 | 0.0006 |
|  | shFUT8 | 2.440±0.2464 |  |
| 10^-6^ | NC | 0.8100±0.1054 | <0.0001 |
|  | shFUT8 | 4.893±0.1504 |  |
| 10^-5^ | NC | 0.9267±0.08083 | 0.0001 |
|  | shFUT8 | 4.713±0.4366 |  |

**Ocn**

| F test to compare variances | | |
| --- | --- | --- |
| sh vs.NC | F | 16.58 |
|  | P value | 0.1138 |
| sh+10^-6^ vs.10^-6^ | F | 3.506 |
|  | P value | 0.4438 |
| sh+10^-5^ vs.10^-5^ | F | 5.239 |
|  | P value | 0.3206 |

| Dex concentration (M) | plasmid | mRNA relative expression | P Value |
| --- | --- | --- | --- |
| 0 | NC | 1.000±0.07000 | 0.0020 |
|  | shFUT8 | 2.213±0.2850 |  |
| 10^-6^ | NC | 0.9267±0.1358 | 0.0001 |
|  | shFUT8 | 3.363±0.2542 |  |
| 10^-5^ | NC | 0.8533±0.1250 | <0.0001 |
|  | shFUT8 | 3.690±0.2862 |  |

**Osx**

| F test to compare variances | | |
| --- | --- | --- |
| sh vs.NC | F | 15.18 |
|  | P value | 0.1236 |
| sh+10^-6^ vs.10^-6^ | F | 12.64 |
|  | P value | 0.1467 |
| sh+10^-5^ vs.10^-5^ | F | 37.68 |
|  | P value | 0.0517 |

| Dex concentration (M) | plasmid | mRNA relative expression | P Value |
| --- | --- | --- | --- |
| 0 | NC | 1.003±0.07572 | 0.0029 |
|  | shFUT8 | 2.147±0.2950 |  |
| 10^-6^ | NC | 1.047±0.1358 | 0.0005 |
|  | shFUT8 | 4.097±0.4826 |  |
| 10^-5^ | NC | 0.8233±0.05132 | <0.0001 |
|  | shFUT8 | 3.823±0.3150 |  |

**RUNX2**

| F test to compare variances | | |
| --- | --- | --- |
| sh vs.NC | F | 15.18 |
|  | P value | 0.1236 |
| sh+10^-6^ vs.10^-6^ | F | 12.64 |
|  | P value | 0.1467 |
| sh+10^-5^ vs.10^-5^ | F | 37.68 |
|  | P value | 0.0517 |

| Dex concentration (M) | plasmid | mRNA relative expression | P Value |
| --- | --- | --- | --- |
| 0 | NC | 1.003±0.07572 | 0.0029 |
|  | shFUT8 | 2.147±0.2950 |  |
| 10^-6^ | NC | 1.047±0.1358 | 0.0005 |
|  | shFUT8 | 4.097±0.4826 |  |
| 10^-5^ | NC | 0.8233±0.05132 | <0.0001 |
|  | shFUT8 | 3.823±0.3150 |  |

**BMP2**

| F test to compare variances | | |
| --- | --- | --- |
| sh vs.NC | F | 6.878 |
|  | P value | 0.2539 |
| sh+10^-6^ vs.10^-6^ | F | 159.6 |
|  | P value | 0.0125 |
| sh+10^-5^ vs.10^-5^ | F | 79.86 |
|  | P value | 0.0247 |

| Dex concentration (M) | plasmid | mRNA relative expression | P Value |
| --- | --- | --- | --- |
| 0 | NC | 1.003±0.07572 | 0.0008 |
|  | shFUT8 | 2.133±0.1986 |  |
| 10^-6^ | NC | 0.6567±0.02887 | 0.0038 |
|  | shFUT8 | 3.977±0.3647 |  |
| 10^-5^ | NC | 0.9533±0.07638 | 0.0123 |
|  | shFUT8 | 4.367±0.6825 |  |

**Figure2B summary:**

| Genes | Dex concentration (M) | plasmid | mRNA relative expression | P Value |
| --- | --- | --- | --- | --- |
| ALP | 0 | NC | 1.003±0.07572 | 0.0006 |
|  |  | shFUT8 | 2.440±0.2464 |  |
|  | 10^-6^ | NC | 0.8100±0.1054 | <0.0001 |
|  |  | shFUT8 | 4.893±0.1504 |  |
|  | 10^-5^ | NC | 0.9267±0.08083 | 0.0001 |
|  |  | shFUT8 | 4.713±0.4366 |  |
| Ocn | 0 | NC | 1.000±0.07000 | 0.0020 |
|  |  | shFUT8 | 2.213±0.2850 |  |
|  | 10^-6^ | NC | 0.9267±0.1358 | 0.0001 |
|  |  | shFUT8 | 3.363±0.2542 |  |
|  | 10^-5^ | NC | 0.8533±0.1250 | <0.0001 |
|  |  | shFUT8 | 3.690±0.2862 |  |
| Osx | 0 | NC | 1.003±0.07572 | 0.0029 |
|  |  | shFUT8 | 2.147±0.2950 |  |
|  | 10^-6^ | NC | 1.047±0.1358 | 0.0005 |
|  |  | shFUT8 | 4.097±0.4826 |  |
|  | 10^-5^ | NC | 0.8233±0.05132 | <0.0001 |
|  |  | shFUT8 | 3.823±0.3150 |  |
| RUNX2 | 0 | NC | 1.003±0.07572 | 0.0029 |
|  |  | shFUT8 | 2.147±0.2950 |  |
|  | 10^-6^ | NC | 1.047±0.1358 | 0.0005 |
|  |  | shFUT8 | 4.097±0.4826 |  |
|  | 10^-5^ | NC | 0.8233±0.05132 | <0.0001 |
|  |  | shFUT8 | 3.823±0.3150 |  |
| BMP2 | 0 | NC | 1.003±0.07572 | 0.0008 |
|  |  | shFUT8 | 2.133±0.1986 |  |
|  | 10^-6^ | NC | 0.6567±0.02887 | 0.0038 |
|  |  | shFUT8 | 3.977±0.3647 |  |
|  | 10^-5^ | NC | 0.9533±0.07638 | 0.0123 |
|  |  | shFUT8 | 4.367±0.6825 |  |

**TGFβ1**

| F test to compare variances | | |
| --- | --- | --- |
| sh vs.NC | F | 14.17 |
|  | P value | 0.1319 |
| sh+10^-6^ vs.10^-6^ | F | 17.71 |
|  | P value | 0.1069 |
| sh+10^-5^ vs.10^-5^ | F | 28.07 |
|  | P value | 0.0688 |

| Dex concentration (M) | plasmid | mRNA relative expression | P Value |
| --- | --- | --- | --- |
| 0 | NC | 1.003±0.07572 | 0.0022 |
|  | shFUT8 | 2.197±0.2850 |  |
| 10^-6^ | NC | 0.6533±0.08083 | 0.0003 |
|  | shFUT8 | 3.087±0.3402 |  |
| 10^-5^ | NC | 0.9333±0.08505 | 0.0002 |
|  | shFUT8 | 4.637±0.4506 |  |

**TGFβ2**

| F test to compare variances | | |
| --- | --- | --- |
| sh vs.NC | F | 10.11 |
|  | P value | 0.1800 |
| sh+10^-6^ vs.10^-6^ | F | 2.461 |
|  | P value | 0.5778 |
| sh+10^-5^ vs.10^-5^ | F | 13.27 |
|  | P value | 0.1402 |

| Dex concentration (M) | plasmid | mRNA relative expression | P Value |
| --- | --- | --- | --- |
| 0 | NC | 1.003±0.1266 | 0.0002 |
|  | shFUT8 | 4.203±0.4027 |  |
| 10^-6^ | NC | 1.567±0.1650 | 0.4738 |
|  | shFUT8 | 1.427±0.2589 |  |
| 10^-5^ | NC | 1.323±0.1474 | 0.0006 |
|  | shFUT8 | 4.520±0.5369 |  |

**TGFβ3**

| F test to compare variances | | |
| --- | --- | --- |
| sh vs.NC | F | 25.92 |
|  | P value | 0.0743 |
| sh+10^-6^ vs.10^-6^ | F | 8.355 |
|  | P value | 0.2138 |
| sh+10^-5^ vs.10^-5^ | F | 5.262 |
|  | P value | 0.3194 |

| Dex concentration (M) | plasmid | mRNA relative expression | P Value |
| --- | --- | --- | --- |
| 0 | NC | 1.003±0.1060 | 0.0002 |
|  | shFUT8 | 0.2233±0.02082 |  |
| 10^-6^ | NC | 0.9133±0.09292 | 0.0002 |
|  | shFUT8 | 0.1333±0.03215 |  |
| 10^-5^ | NC | 1.793±0.1504 | 0.6795 |
|  | shFUT8 | 1.890±0.3451 |  |

**Smad2**

| F test to compare variances | | |
| --- | --- | --- |
| sh vs.NC | F | 15.61 |
|  | P value | 0.1204 |
| sh+10^-6^ vs.10^-6^ | F | 45.69 |
|  | P value | 0.0428 |
| sh+10^-5^ vs.10^-5^ | F | 8.158 |
|  | P value | 0.2184 |

| Dex concentration (M) | plasmid | mRNA relative expression | P Value |
| --- | --- | --- | --- |
| 0 | NC | 1.003±0.06028 | 0.0041 |
|  | shFUT8 | 1.840±0.2381 |  |
| 10^-6^ | NC | 0.5867±0.08622 | 0.0031 |
|  | shFUT8 | 6.120±0.5828 |  |
| 10^-5^ | NC | 1.090±0.1637 | 0.0003 |
|  | shFUT8 | 4.557±0.1637 |  |

**Smad3**

| F test to compare variances | | |
| --- | --- | --- |
| sh vs.NC | F | 4.000 |
|  | P value | 0.4000 |
| sh+10^-6^ vs.10^-6^ | F | 43.89 |
|  | P value | 0.0446 |
| sh+10^-5^ vs.10^-5^ | F | 146.7 |
|  | P value | 0.0135 |

| Dex concentration (M) | plasmid | mRNA relative expression | P Value |
| --- | --- | --- | --- |
| 0 | NC | 1.003±0.1026 | 0.0024 |
|  | shFUT8 | 1.913±0.2053 |  |
| 10^-6^ | NC | 0.6867±0.04509 | 0.0063 |
|  | shFUT8 | 2.693±0.2987 |  |
| 10^-5^ | NC | 0.8333±0.05508 | 0.0038 |
|  | shFUT8 | 6.897±0.6671 |  |

**Smad4**

| F test to compare variances | | |
| --- | --- | --- |
| sh vs.NC | F | 9.241 |
|  | P value | 0.1953 |
| sh+10^-6^ vs.10^-6^ | F | 1.137 |
|  | P value | 0.9361 |
| sh+10^-5^ vs.10^-5^ | F | 10.91 |
|  | P value | 0.1679 |

| Dex concentration (M) | plasmid | mRNA relative expression | P Value |
| --- | --- | --- | --- |
| 0 | NC | 1.007±0.1159 | 0.0002 |
|  | shFUT8 | 3.977±0.3523 |  |
| 10^-6^ | NC | 1.577±0.1553 | 0.0582 |
|  | shFUT8 | 1.253±0.1457 |  |
| 10^-5^ | NC | 1.357±0.1457 | 0.0005 |
|  | shFUT8 | 4.330±0.4814 |  |

**TGFβRI**

| F test to compare variances | | |
| --- | --- | --- |
| sh vs.NC | F | 17.74 |
|  | P value | 0.1067 |
| sh+10^-6^ vs.10^-6^ | F | 73.00 |
|  | P value | 0.0270 |
| sh+10^-5^ vs.10^-5^ | F | 5.232 |
|  | P value | 0.3209 |

| Dex concentration (M) | plasmid | mRNA relative expression | P Value |
| --- | --- | --- | --- |
| 0 | NC | 1.003±0.1060 | 0.0002 |
|  | shFUT8 | 0.2067±0.02517 |  |
| 10^-6^ | NC | 0.8867±0.09866 | 0.0053 |
|  | shFUT8 | 0.1433±0.01155 |  |
| 10^-5^ | NC | 1.790±0.1552 | 0.7081 |
|  | shFUT8 | 1.880±0.3551 |  |

**TGFβRII**

| F test to compare variances | | |
| --- | --- | --- |
| sh vs.NC | F | 16.00 |
|  | P value | 0.1176 |
| sh+10^-6^ vs.10^-6^ | F | 5.018 |
|  | P value | 0.3323 |
| sh+10^-5^ vs.10^-5^ | F | 95.14 |
|  | P value | 0.0208 |

| Dex concentration (M) | plasmid | mRNA relative expression | P Value |
| --- | --- | --- | --- |
| 0 | NC | 1.000±0.07000 | 0.0018 |
|  | shFUT8 | 2.230±0.2800 |  |
| 10^-6^ | NC | 0.7067±0.08622 | <0.0001 |
|  | shFUT8 | 3.870±0.1931 |  |
| 10^-5^ | NC | 1.003±0.08622 | 0.0034 |
|  | shFUT8 | 9.037±0.8410 |  |

| Genes | Dex concentration (M) | plasmid | mRNA relative expression | P Value |
| --- | --- | --- | --- | --- |
| TGFβ1 | 0 | NC | 1.003±0.07572 | 0.0022 |
|  |  | shFUT8 | 2.197±0.2850 |  |
|  | 10^-6^ | NC | 0.6533±0.08083 | 0.0003 |
|  |  | shFUT8 | 3.087±0.3402 |  |
|  | 10^-5^ | NC | 0.9333±0.08505 | 0.0002 |
|  |  | shFUT8 | 4.637±0.4506 |  |
| TGFβ2 | 0 | NC | 1.003±0.1266 | 0.0002 |
|  |  | shFUT8 | 4.203±0.4027 |  |
|  | 10^-6^ | NC | 1.567±0.1650 | 0.4738 |
|  |  | shFUT8 | 1.427±0.2589 |  |
|  | 10^-5^ | NC | 1.323±0.1474 | 0.0006 |
|  |  | shFUT8 | 4.520±0.5369 |  |
| TGFβ3 | 0 | NC | 1.003±0.1060 | 0.0002 |
|  |  | shFUT8 | 0.2233±0.02082 |  |
|  | 10^-6^ | NC | 0.9133±0.09292 | 0.0002 |
|  |  | shFUT8 | 0.1333±0.03215 |  |
|  | 10^-5^ | NC | 1.793±0.1504 | 0.6795 |
|  |  | shFUT8 | 1.890±0.3451 |  |
| Smad2 | 0 | NC | 1.003±0.06028 | 0.0041 |
|  |  | shFUT8 | 1.840±0.2381 |  |
|  | 10^-6^ | NC | 0.5867±0.08622 | 0.0031 |
|  |  | shFUT8 | 6.120±0.5828 |  |
|  | 10^-5^ | NC | 1.090±0.1637 | 0.0003 |
|  |  | shFUT8 | 4.557±0.1637 |  |
| Smad3 | 0 | NC | 1.003±0.1026 | 0.0024 |
|  |  | shFUT8 | 1.913±0.2053 |  |
|  | 10^-6^ | NC | 0.6867±0.04509 | 0.0063 |
|  |  | shFUT8 | 2.693±0.2987 |  |
|  | 10^-5^ | NC | 0.8333±0.05508 | 0.0038 |
|  |  | shFUT8 | 6.897±0.6671 |  |
| Smad4 | 0 | NC | 1.007±0.1159 | 0.0002 |
|  |  | shFUT8 | 3.977±0.3523 |  |
|  | 10^-6^ | NC | 1.577±0.1553 | 0.0582 |
|  |  | shFUT8 | 1.253±0.1457 |  |
|  | 10^-5^ | NC | 1.357±0.1457 | 0.0005 |
|  |  | shFUT8 | 4.330±0.4814 |  |
| TGFβRI | 0 | NC | 1.003±0.1060 | 0.0002 |
|  |  | shFUT8 | 0.2067±0.02517 |  |
|  | 10^-6^ | NC | 0.8867±0.09866 | 0.0053 |
|  |  | shFUT8 | 0.1433±0.01155 |  |
|  | 10^-5^ | NC | 1.790±0.1552 | 0.7081 |
|  |  | shFUT8 | 1.880±0.3551 |  |
| TGFβRII | 0 | NC | 1.000±0.07000 | 0.0018 |
|  |  | shFUT8 | 2.230±0.2800 |  |
|  | 10^-6^ | NC | 0.7067±0.08622 | <0.0001 |
|  |  | shFUT8 | 3.870±0.1931 |  |
|  | 10^-5^ | NC | 1.003±0.08622 | 0.0034 |
|  |  | shFUT8 | 9.037±0.8410 |  |

**Figure 4D:**

**TGFβ1 binding assay**

|  | F | P value |
| --- | --- | --- |
| Dexconcentration x overexpression | 3.875 | 0.0503 |
| Dexconcentration | 84.15 | <0.0001 |
| overexpression | 979.6 | <0.0001 |

| Dexconcentration (M) | plasmid | mRNA relative expression | P Value |
| --- | --- | --- | --- |
| 0 | NC | 5.853±0.3921 | <0.0001 |
|  | shFUT8 | 16.71±0.3623 |  |
| 10^-6^ | NC | 3.433±0.5263 | <0.0001 |
|  | shFUT8 | 12.44±1.0422 |  |
| 10^-5^ | NC | 1.980±0.3804 | <0.0001 |
|  | shFUT8 | 11.05±0.8769 |  |
