# Supplementary figures and images for: Overexpression of fucosyltransferase 8 reverses the inhibitory effect of high-dose dexamethasone on osteogenic response of MC3T3-E1 preosteoblasts
Source: PeerJ. 2021 Dec 9;9:e12380. doi: 10.7717/peerj.12380 (PMC8667747; doi:10.7717/peerj.12380)

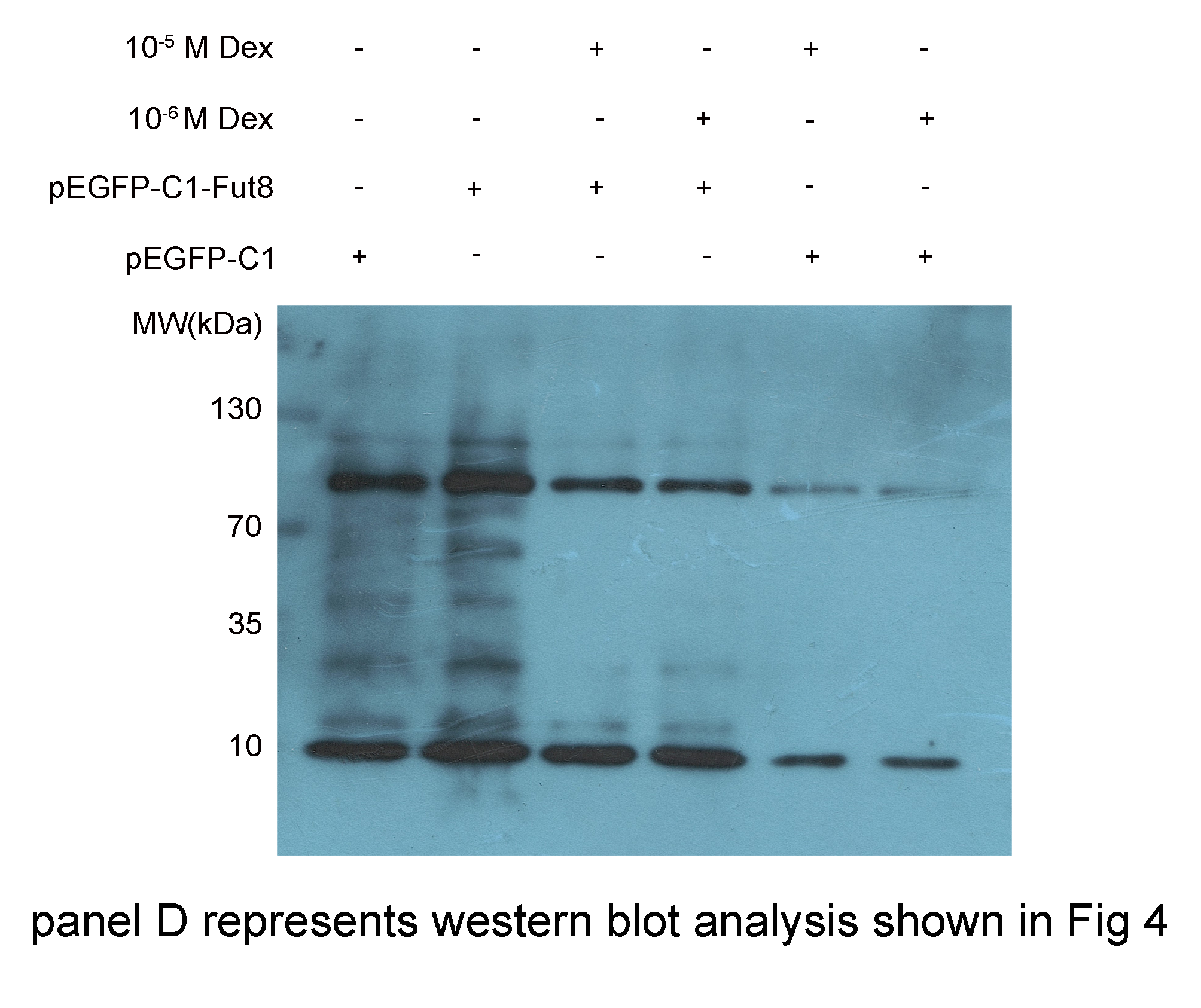

Supplement: Supplemental Information 1 [file peerj-09-12380-s001.zip › Supplementary Materials 20210719/Original Blots/Lectin blot (cell).tif]

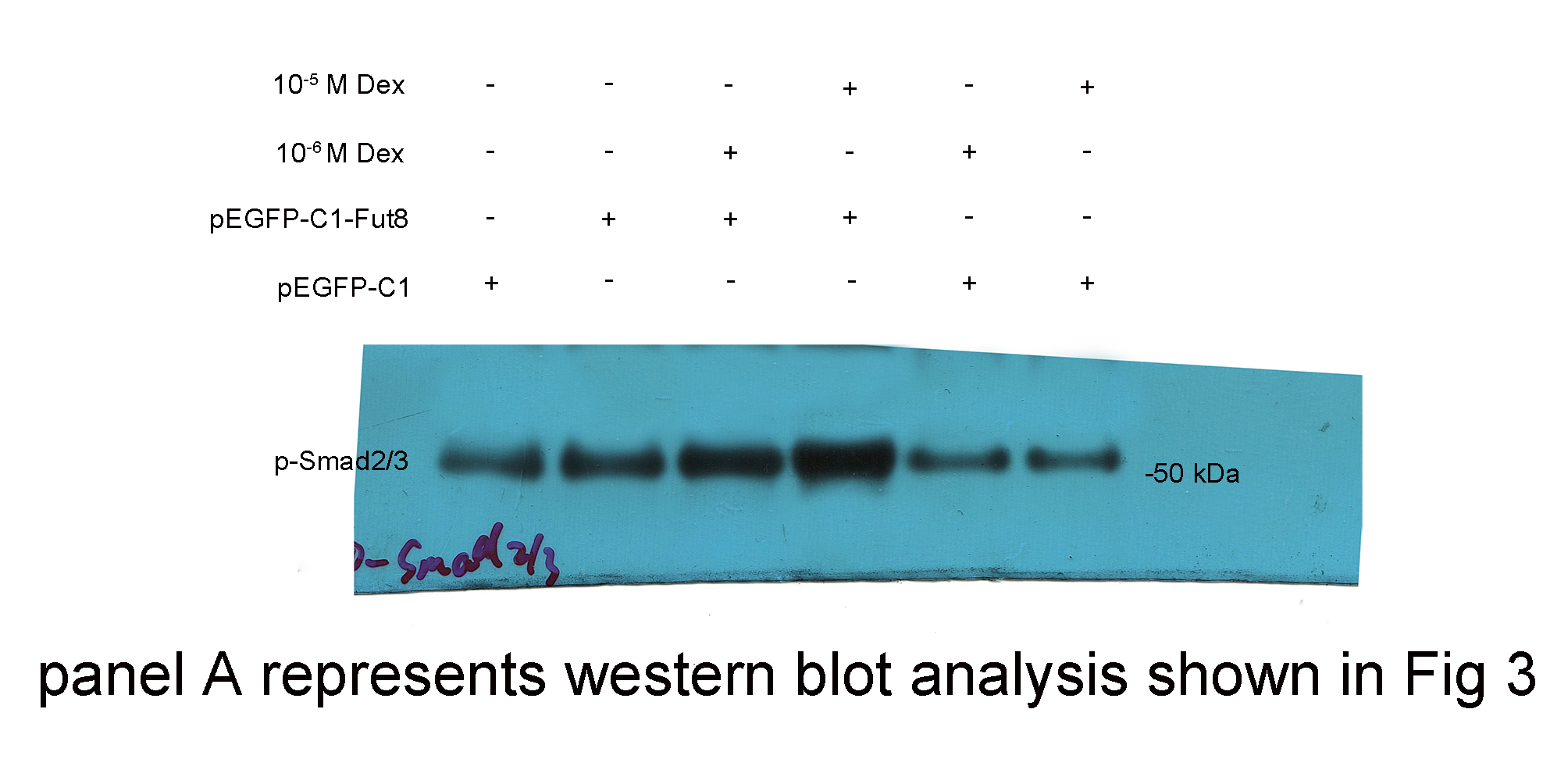

Supplement: Supplemental Information 1 [file peerj-09-12380-s001.zip › Supplementary Materials 20210719/Original Blots/p-smad23.tif]

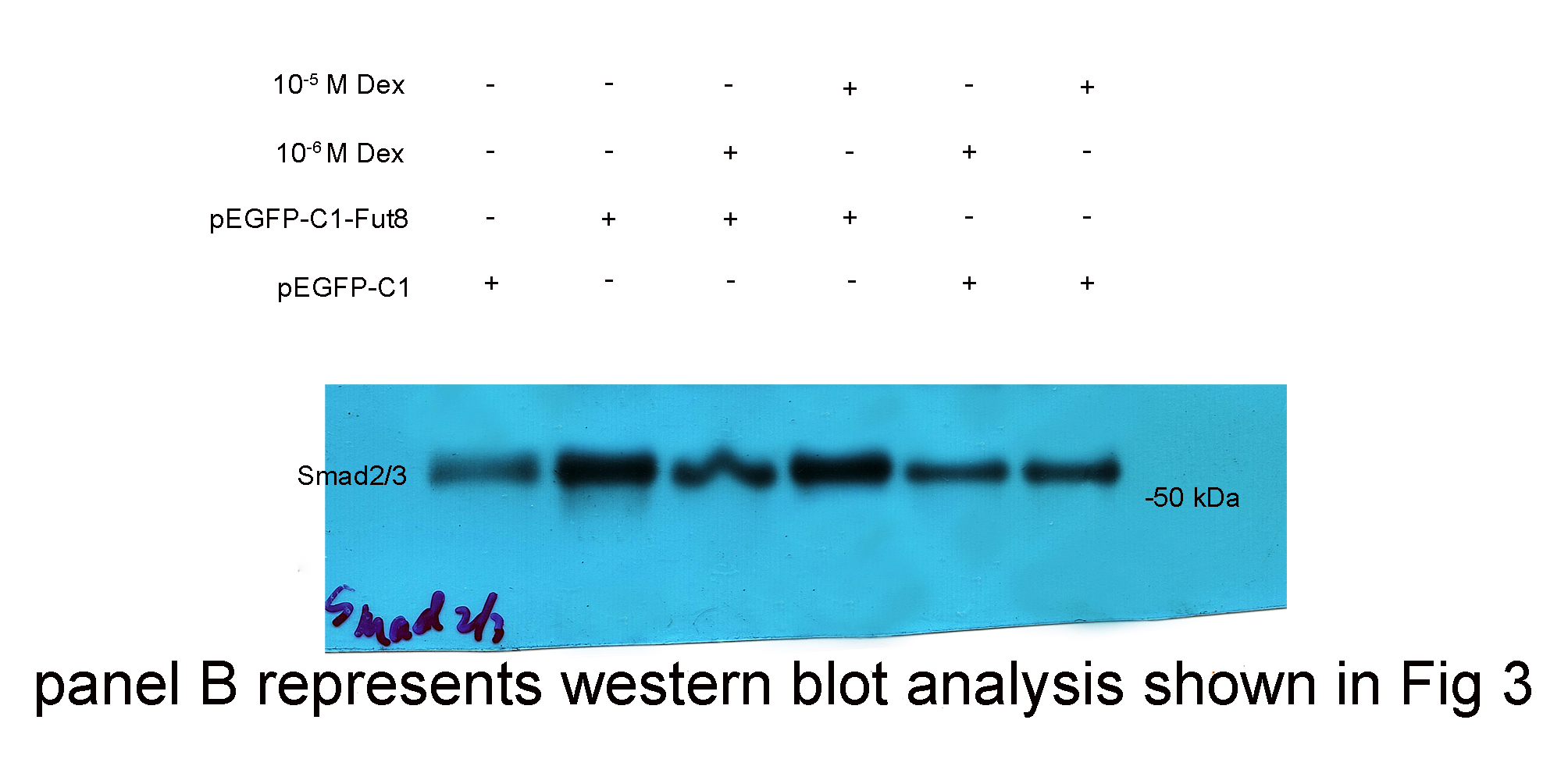

Supplement: Supplemental Information 1 [file peerj-09-12380-s001.zip › Supplementary Materials 20210719/Original Blots/smad23.tif]

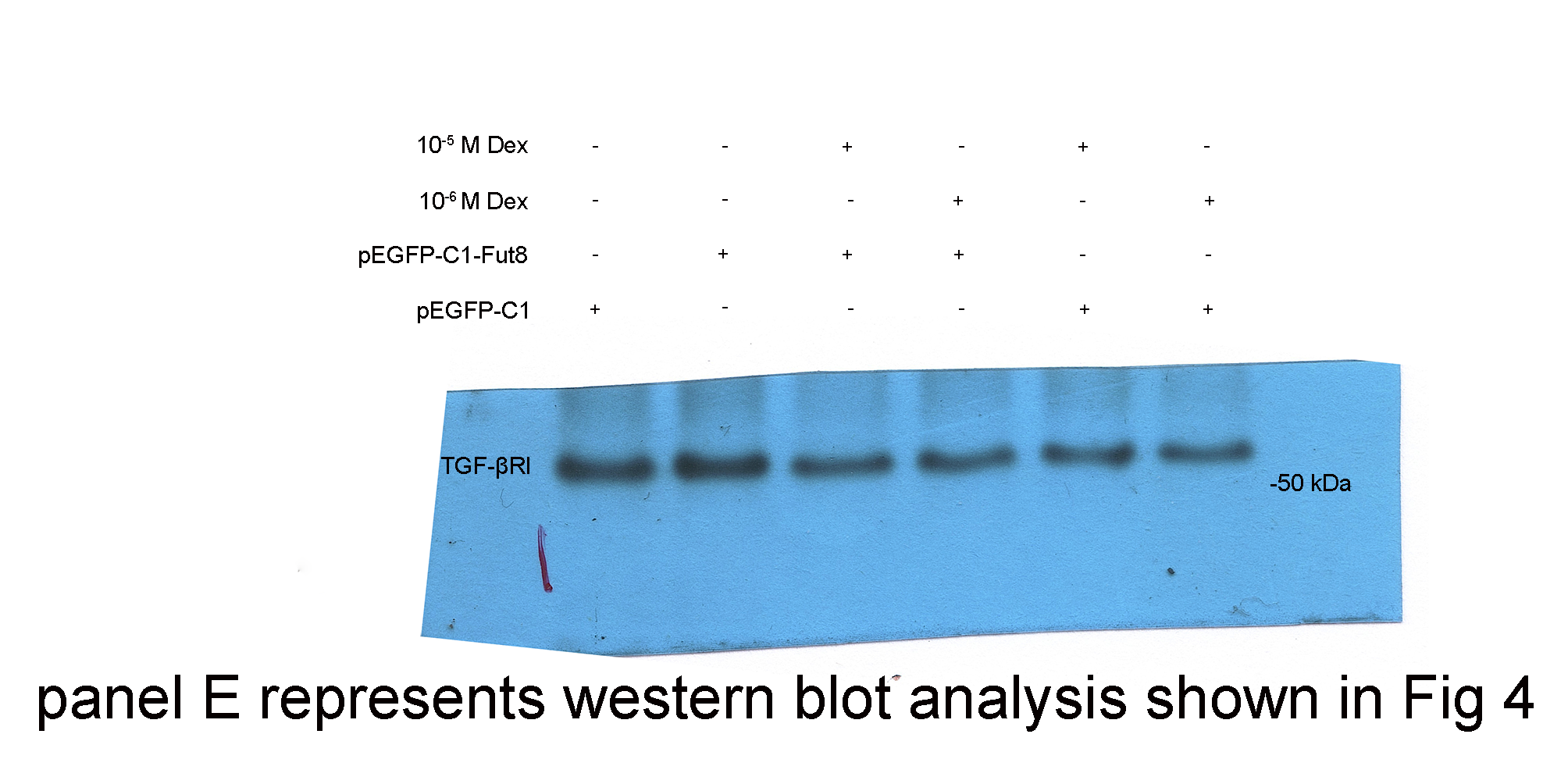

Supplement: Supplemental Information 1 [file peerj-09-12380-s001.zip › Supplementary Materials 20210719/Original Blots/TGFa┬RI.tif]

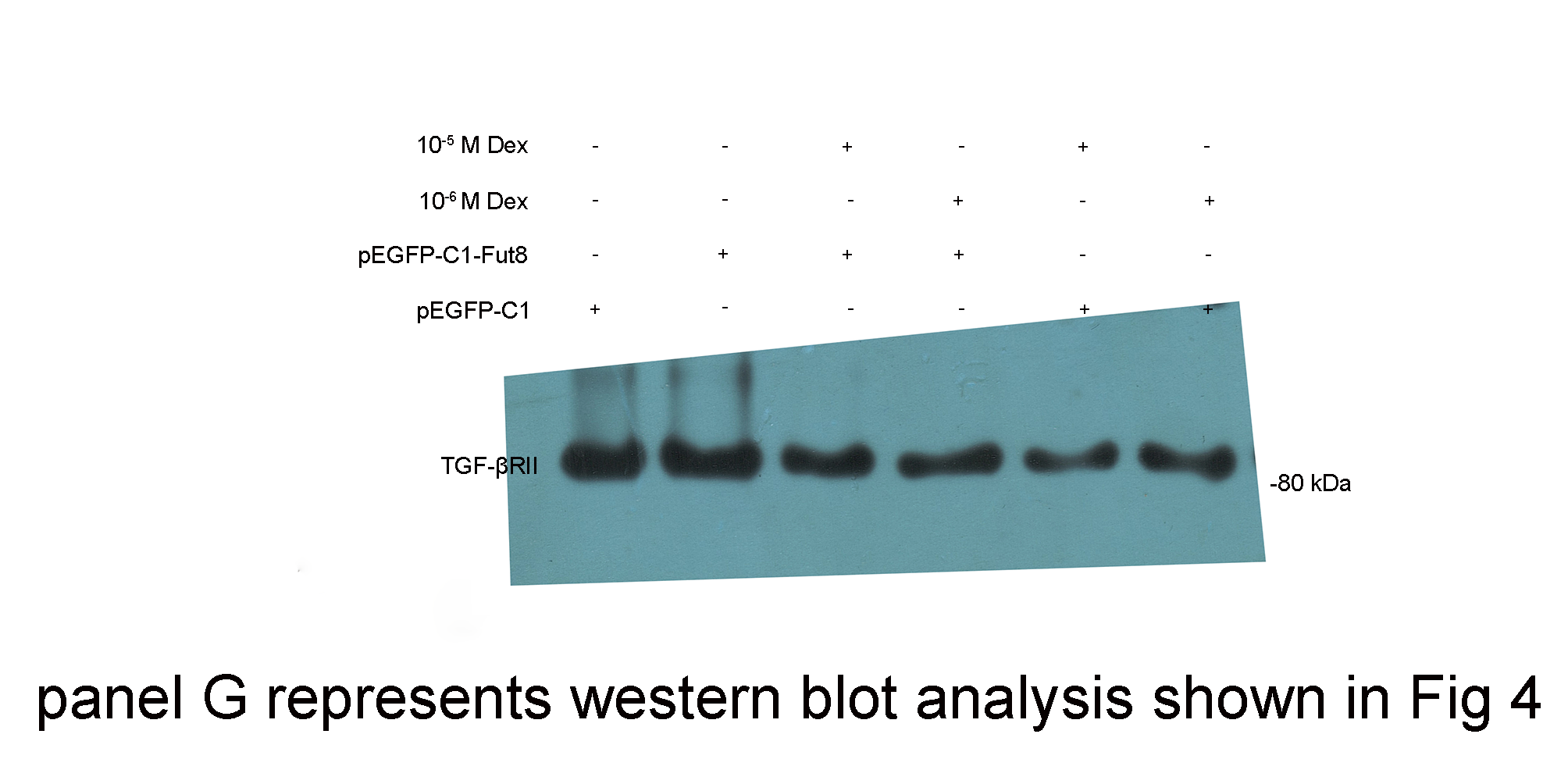

Supplement: Supplemental Information 1 [file peerj-09-12380-s001.zip › Supplementary Materials 20210719/Original Blots/TGFa┬RII.tif]

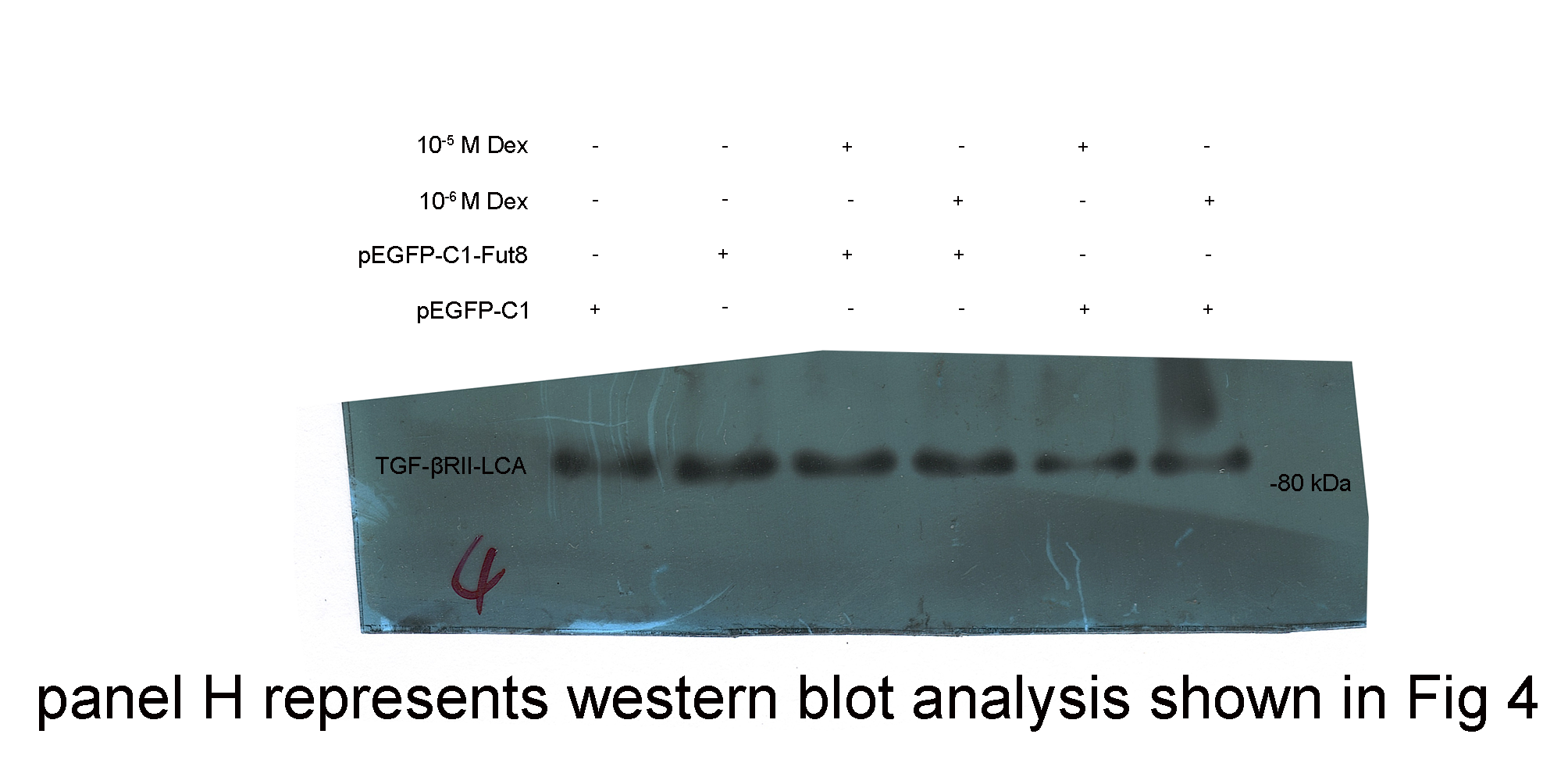

Supplement: Supplemental Information 1 [file peerj-09-12380-s001.zip › Supplementary Materials 20210719/Original Blots/TGFa┬RII-LCA.tif]

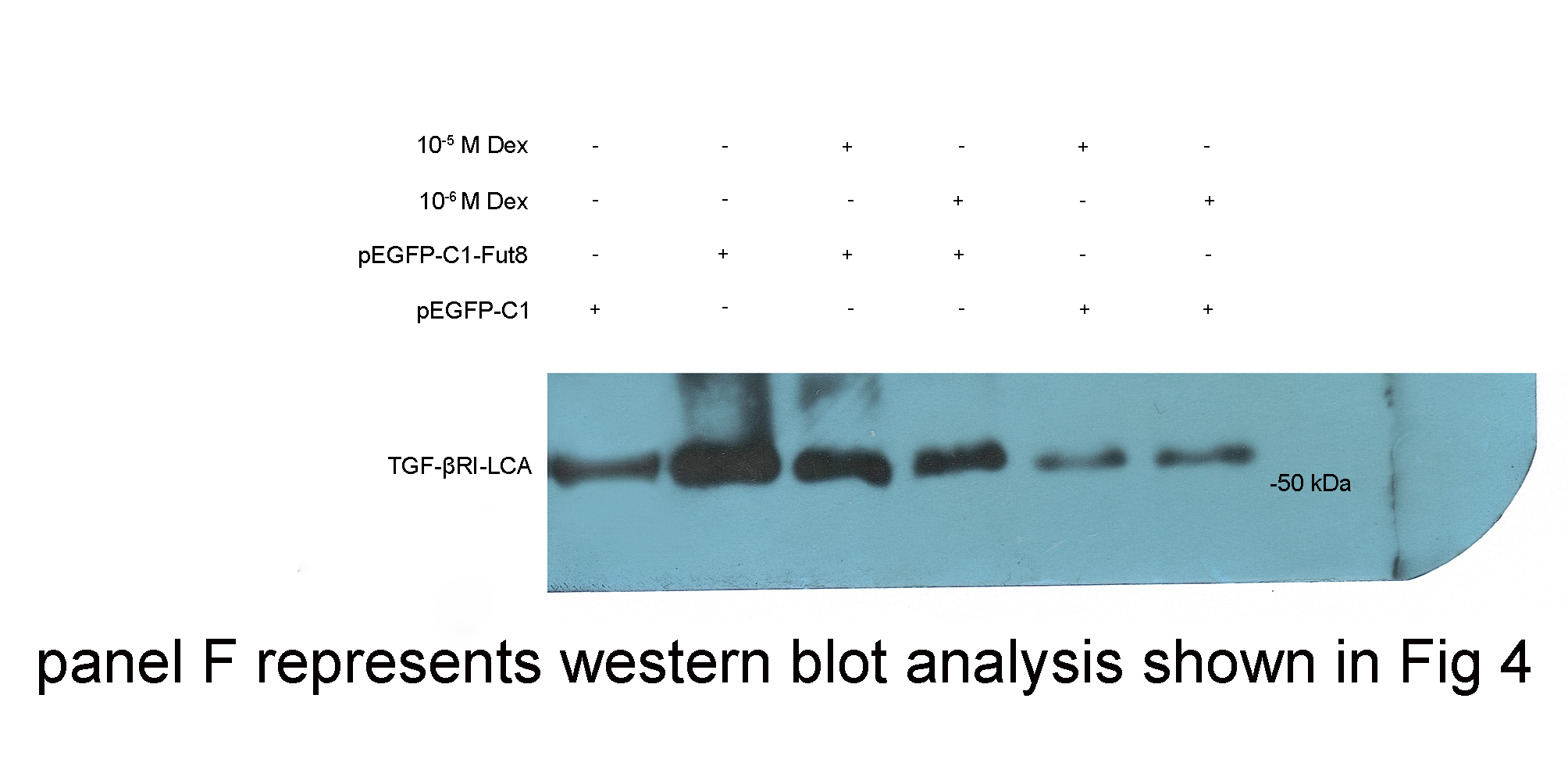

Supplement: Supplemental Information 1 [file peerj-09-12380-s001.zip › Supplementary Materials 20210719/Original Blots/TGFa┬RI-LCA.tif]

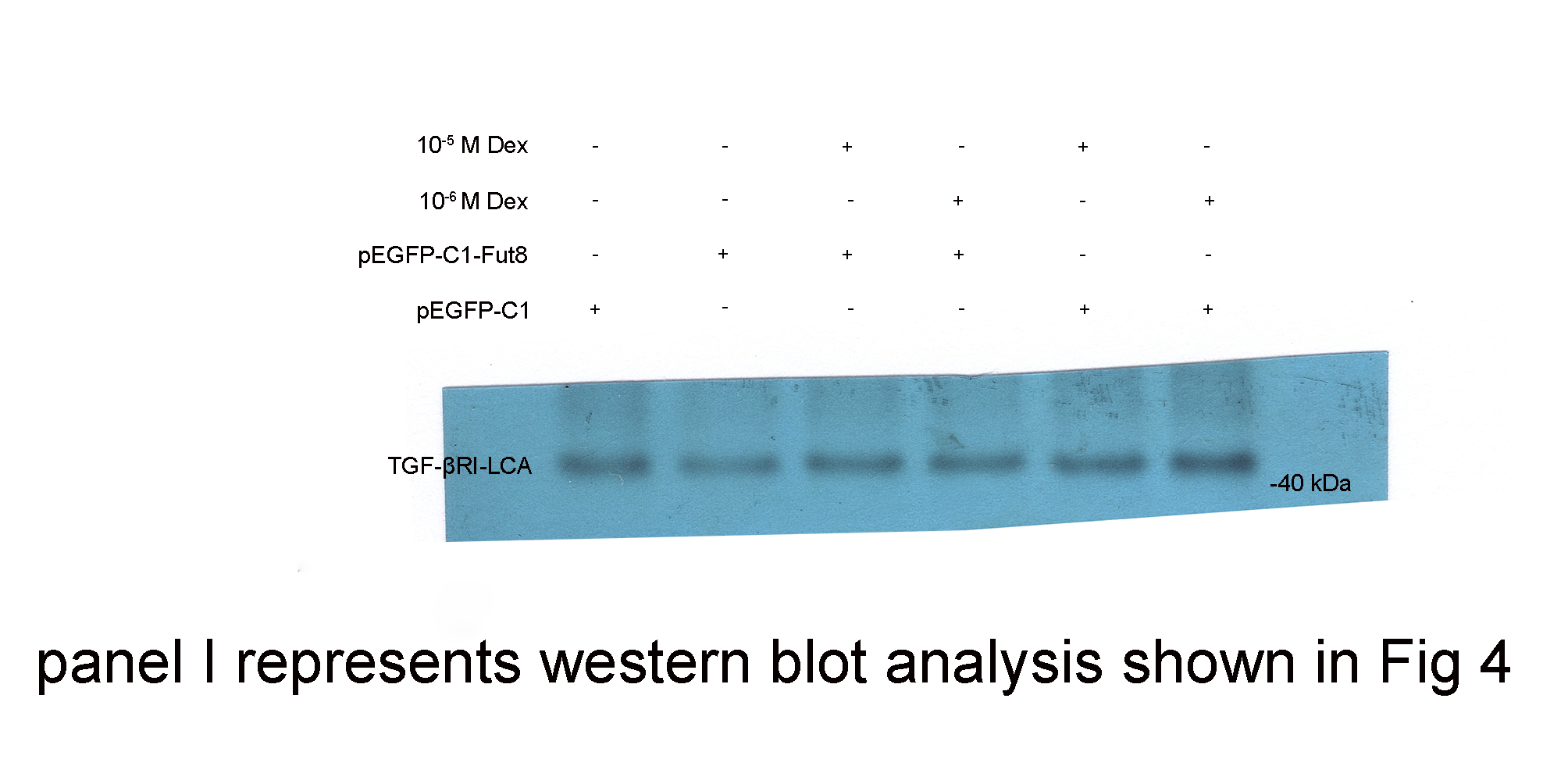

Supplement: Supplemental Information 1 [file peerj-09-12380-s001.zip › Supplementary Materials 20210719/Original Blots/a┬-actin (Lecin).tif]

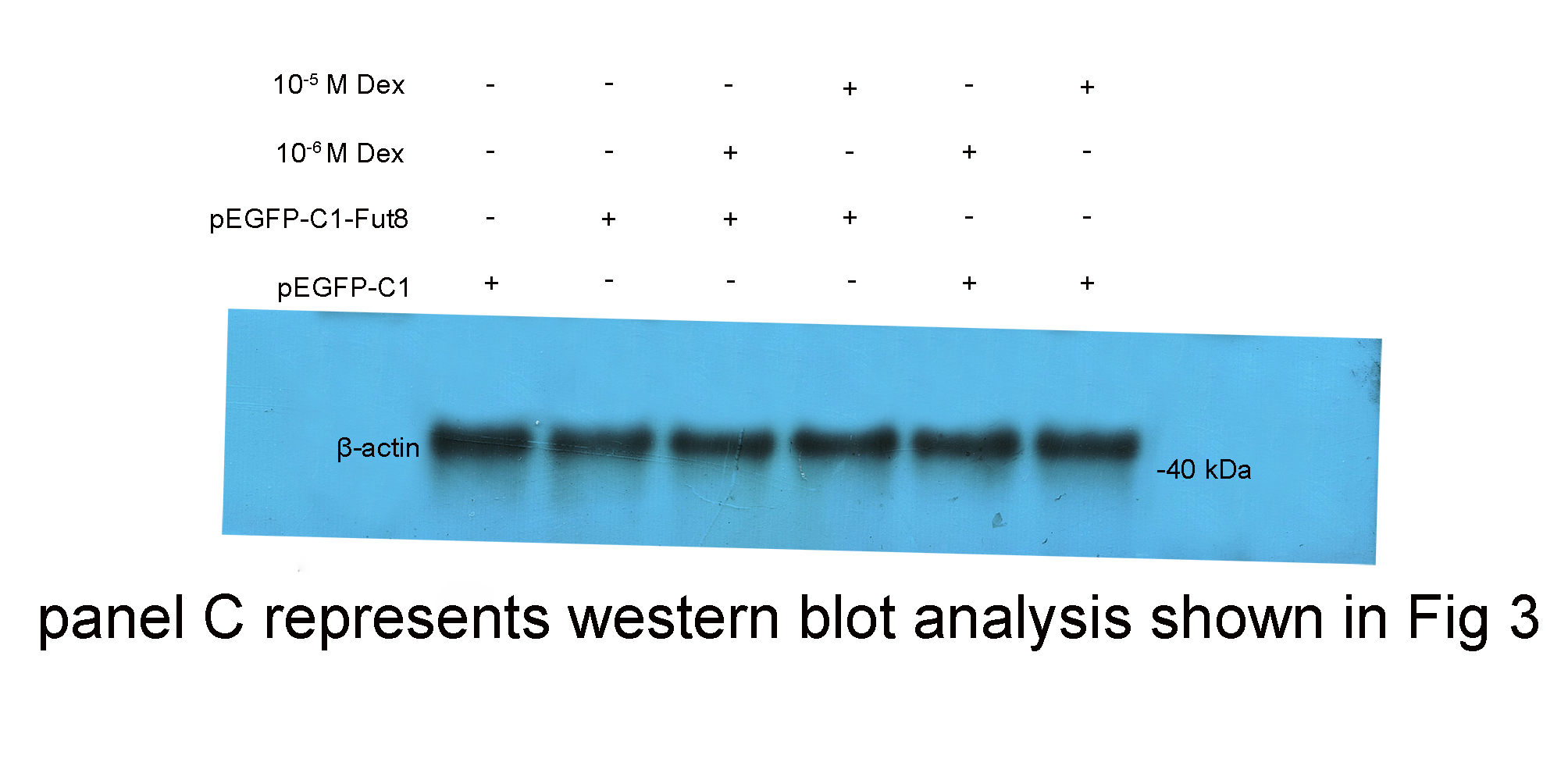

Supplement: Supplemental Information 1 [file peerj-09-12380-s001.zip › Supplementary Materials 20210719/Original Blots/a┬-actin.tif]
